# Evaluation of calcium carbide residue and fly ash as sustainable binders for environmentally friendly loess soil stabilization

**DOI:** 10.1038/s41598-024-51326-x

**Published:** 2024-01-05

**Authors:** Phongthorn Julphunthong, Panuwat Joyklad, Papantasorn Manprom, Thanakorn Chompoorat, Martin-Tchingnabé Palou, Tawat Suriwong

**Affiliations:** 1 Department of Civil Engineering, Faculty of Engineering, Naresuan University, Phitsanulok, 65000 Thailand; 2 Present Address: Research Center for Academic Excellence in Applied Physics, Faculty of Science, Naresuan University, Phitsanulok, 65000 Thailand; 3 Department of Civil and Environmental Engineering, Faculty of Engineering, Srinakharinwirot University, Nakhon Nayok, 26120 Thailand; 4 Department of Civil Engineering, School of Engineering, University of Phayao, Phayao, 56000 Thailand; 5 Institute of Construction and Architecture, Slovak Academy of Sciences, Dúbravská Cesta 9, Bratislava, 845 03 Slovak Republic; 6 School of Renewable Energy and Smart Grid Technology, Naresuan University, Phitsanulok, 65000 Thailand

**Keywords:** Civil engineering, Structural materials

## Abstract

The incorporation of waste materials into cementitious binders serves as a strategy to diminish waste volume and lower carbon emissions. This study presents an in-depth evaluation of calcium carbide residue and coal fly ash as alternative binders. The assessment of raw materials emphasized their chemical composition and potential for pozzolanic reactions. Based on these factors, the optimal ratio of Ca/(SiO_2_ + Al_2_O_3_) in the raw materials was determined to be 1.5. Therefore, this study was designed to vary the raw material composition with a CaO/(SiO_2_ + Al_2_O_3_) ratio ranging from 1.7 to 0.9. Upon investigating the effect of the raw material proportion on the compressive strength of pastes and mortars, the composition yielding the highest compressive strength was selected for its potential application as a stabilizer for loess soil. A mixture of calcium carbide residue and coal fly ash with a Ca/(SiO_2_ + Al_2_O_3_) ratio of 1.5 resulted in the highest compressive strength at long curing periods in both pastes and mortars. Mineralogical and microstructural analyses revealed several products, beyond those formed from the pozzolanic reactions, that occurred and enhanced the compressive strength of samples. The highest performing mixture of carbide residue and coal fly ash was then used to stabilize loess soil at 10–25 wt%. The unconfined compressive strength, along with mass and strength loss due to wetting and drying cycles, was also studied. It was observed that the unconfined compressive strength of the stabilized soils remained consistent after six wet-dry cycles but decreased after twelve cycles due to microcracks. The findings suggest that carefully designed mixtures based on the chemical interactions of calcium carbide residue and coal fly ash can offer a sustainable, efficient approach for soil stabilization, potentially revolutionizing construction practices.

## Introduction

In the quest for sustainable construction, the industry is actively seeking supplementary cementing materials (SCMs) as alternatives to Ordinary Portland Cement (OPC). The reasons are threefold: environmental impact, resource conservation, and cost-effectiveness. OPC production contributes about 8% of the world’s CO_2_ emissions, hence a more environmentally friendly option is required. SCMs reduce waste and conserve non-renewable resources because they are made from industrial byproducts including fly ash, slag, and silica fume. Moreover, SCMs enhance concrete durability, lower maintenance costs, and reduce overall project expenses, making them a viable, eco-friendly alternative for the future of construction. Over the past few years, the concrete sector has undertaken several measures to minimize its carbon footprint. These measures include reducing the consumption of raw materials, improving energy efficiency in kilns, transitioning from fossil fuels to renewable sources, and incorporating supplementary cementitious materials, also known as pozzolanic materials, such as coal fly ash (FA), ground granulated blast furnace slag (GGBFS), rice husk ash (RHA), circulating fluidized bed combustion fly ash (CFA), and locally sourced materials. The pozzolanic materials can undergo a reaction with calcium hydroxide during cement hydration, leading to the generation of extra calcium silicate hydrates (C–S–H). As a consequence, they acquire properties that are comparable to those exhibited by Portland cement. Incorporating these materials into concrete can be a promising strategy to enhance its sustainability.

Calcium carbide residue (CCR) is a by-product resulting from the acetylene manufacturing process, primarily consisting of calcium portlandite and calcite^1,2^. The annual production of calcium carbide residue amounts to about 28 million tons, yet its utilization rate stands at a mere 55%^3^. The topic of interest involves the possibility of replacing ordinary Portland cement with a mixture of CCR waste and fly ash as cementing materials. This is due to the potential reaction between portlandite present in CCR and the amorphous silica or alumina in fly ash, resulting in the formation of reaction products like calcium silicate hydrate and calcium aluminate hydrate. Numerous studies have investigated the individual and combined effects of CCR and FA as SCMs in cement and concrete applications^4–10^. These investigations have revealed that CCR and FA can effectively improve the mechanical properties, durability, and long-term performance. Additionally, the usage of CCR and FA can minimize the heat of hydration, lowering the risk of thermal cracking in large concrete constructions. The utilization of CCR and FA in concrete mixtures contributes to a reduction in the heat of hydration, which effectively lowers the risk of thermal cracking in extensive concrete structures. Furthermore, the incorporation of these materials can strengthen the resistance of concrete to chemical attack, such as sulfate and chloride ingress, which is crucial for structures exposed to aggressive environments.

The successful implementation of CCR and FA as cementing materials is contingent upon a comprehensive understanding of their chemical and physical properties, as well as their interactions with other constituents in the cementitious system. The optimal combination of CCR and FA depends on factors such as their chemical composition, fineness, and the desired performance characteristics of the resulting cementitious material. However, several previous investigations have involved mixing CCR and FA to apply as a cementing material, using trial and error to design the proportion of CCR and FA required to achieve the highest compressive strength paste and mortar^7,11–15^. Since the main reactions that produce a cementitious binder from the mixture of CCR and FA are the pozzolanic reactions between portlandite and silica or alumina, the question is whether it is possible to estimate the mixing proportion of raw CCR and FA that provides the best performance based on their chemical composition.

The utilization of various waste materials as binders to substitute cement is an application gaining traction in soil stabilization, as evidenced by several previous reports^16–19^. Jalal et al. demonstrated the use of sugarcane bagasse ash and waste marble dust as stabilizers for treating expansive soils^20^. Their test results suggested significant improvements in several engineering properties, including plasticity, compactability, swell potential, and strength characteristics. A particularly challenging type of soil in engineering applications is loess, a quaternary continental sediment primarily composed of fine particles. It is characterized by a structure with large pores and weak cementation^21^. Loess soil exhibits significant strength and behaves like soft rock when dry due to its fissured structure. However, its strength decreases with changes in moisture content. Dry–wet cycles, influenced by factors like rainfall and irrigation, can lead to structural weakening, increased porosity, and reduced shear strength. To address the issue of strength loss in loess soil, adding cement is a commonly employed and effective method. Cement stabilization involves mixing Portland cement into the soil, which reacts with water to form cementitious compounds that bind the soil particles together. Additionally, alternative binders such as lime, fly ash, or industrial by-products can be combined with cement and applied as stabilizers, as reported in several previous investigations^22–24^. Gu et al. investigated the effects of cement, fly ash, phosphogypsum, and quicklime on loess stabilization for self-compacting rammed earth construction^25^. Their experimental results revealed that samples with a mix proportion of 100% loess, 10% cement, 3% phosphogypsum, 20% fly ash, and 8% quicklime achieved a high compressive strength of 18.64 MPa at 3 days.

Therefore, this study attempts to investigate cement-free binders utilizing CCR and FA as cementing materials. In the first step, the raw materials were thoroughly examined for their chemical composition, physical properties, and microstructure. Afterward, their chemical composition and potential pozzolanic reactions were used to design several mixture fractions of CCR and FA powders based on the Ca/(SiO_2_ + Al_2_O_3_) ratio. The performance of CCR-FA binders was evaluated through the compressive strength of pastes and mortars, along with morphology and phase formation analysis results. The CCR-FA mixture fraction that exhibited the highest compressive strength was selected as a binder to demonstrate its efficiency as a stabilizer for loess soil. The dry soil was mixed with the stabilizer at concentrations between 10 and 25% of dry soil and compacted at the optimum moisture content to achieve a density within the range of 95% to 105% of the maximum dry density. The compacted soils were tested for unconfined compressive strength at various curing ages. Moreover, this work extends to study mass loss and strength loss due to wetting and drying cycles, which are distinct behavioral problems of loess soils.

## Materials and methods

### Materials

**Figure 1:**
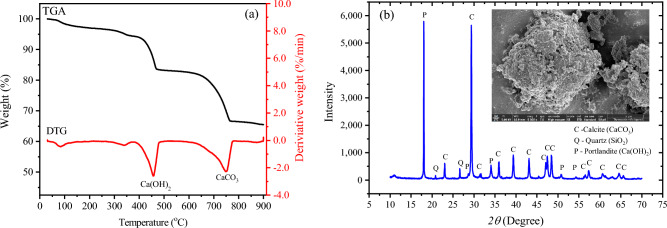
(**a**) TGA-DTG and (**b**) XRD patterns of CCR (inset: SEM image of CCR).

In this investigation, fly ash (FA) and calcium carbide residue (CCR) were both used as binder ingredients. The CCR waste is a by-product obtained during the acetylene gas manufacturing process in Samut Sakhon province, Thailand. To prepare the CCR, it underwent a drying process at 105 °C for 24 h in an oven, then grinding into a fine powder and sieving using a 0.075 mm sieve^2^. The chemical composition of the raw materials used in this work was determined through the Energy-dispersive X-ray fluorescence (EDXRF) technique, and the test results suggest that the main chemical component of CCR is calcium oxide, as indicated in Table 1. The CCR presented a specific gravity of 2.56 and a specific surface area of 218.80 m^2^/kg. Figure 1 (a) presents the Thermogravimetric–Differential Thermal Analysis (TGA-DTA) curves of CCR, indicating the weight loss of the samples with increasing temperature. The first derivative of the TGA-DTG curves was also analyzed to better understand the thermal response of the materials. Two main stages of weight loss were observed: the first stage occurred from 375 °C to 495 °C, likely due to the decomposition of Ca(OH)_2_ in CCR powders^26^, while the second stage occurred between 625 °C and 775 °C and was attributed to the decarbonation of calcite^27^. The phase content of CCR was determined through analysis of X-ray diffraction patterns using the Rietveld refinement technique, as illustrated in Fig. 1b and detailed in Table 2. The predominant peaks of calcite (CaCO_3_) and portlandite (Ca(OH)_2_) were observed, with the phase content suggesting that CCR consists of 66 wt% calcite and 32 wt% portlandite. The SEM image of CCR exhibited irregular shapes and heterogeneous morphology, as shown in the inset figure of Fig. 1b. This is characterized by a mixture of large and small particles, indicative of a broad size distribution. The particles possess a rough, uneven surface, with some exhibiting angular shapes and others appearing more rounded.

**Table 1:** Chemical composition and physical properties of raw materials.

Sample description	CCR	FA	Loess
Chemical composition (wt%)			
SiO_2_	1.84	35.89	56.24
Al_2_O_3_	–	19.62	24.99
Fe_2_O_3_	–	10.64	12.38
CaO	96.70	16.08	0.95
MgO	0.13	2.45	–
K_2_O	0.38	1.05	3.27
Na_2_O	–	2.31	–
SO_3_	0.85	4.08	0.41
Physical properties			
Specific gravity	2.56	2.18	2.70
Median particle size, D_50_ (µm)	63.80	63.60	51.82
Uniformity	1.06	1.59	2.72
Specific surface area (m^2^/kg)	218.80	268.17	222.43
Liquid limit (%)	–	–	18.98
Plastic limit (%)	–	–	NP
Maximum dry density (kg/m^3^)	–	–	1980
Optimum Moisture Content (%)	–	–	9.43
Soil classification (USCS)	–	–	SM

**Table 2:** Quantitative phase analysis of raw materials derived from X-ray Diffraction patterns using the Rietveld refinement method.

Component	CCR	FA	Loess
	Phase composition (wt%)		
Calcite	65.75		
Portlandite	32.02		
Lime (CaO)		1.56	
Quartz	2.23	18.14	91.12
Anhydrite		8.32	
Mullite		7.98	
Iron oxide		1.44	
Kaolinite			7.87
Amorphous		57.16	

The used fly ash is obtained from lignite fly ash at the Mae Moh power plant located in Lampang province, Thailand. According to ASTM C618 standards, the chemical analysis of the fly ash was performed^28^, and the results are summarized in Table 1. The chemical analysis revealed that the sum of SiO_2_, Al_2_O_3_, and Fe_2_O_3_ accounted for 66.15% of the total composition, which categorizes this fly ash as class C. Moreover, the sulfur trioxide (SO_3_) content is determined to be 4.08 wt%, which is marginally below the maximum threshold prescribed by ASTM C618 standard. This standard recommends that the SO_3_ content should not surpass 5.0 wt% for class C fly ash. It should be noted that the SO_3_ present in the FA might undergo a chemical reaction with calcium hydroxide in the CCR. This interaction is believed to instigate a deleterious effect, potentially leading to the development of cracks and subsequent expansion in the composition of pastes, mortars, and other stabilized materials^29^.

**Figure 2:**
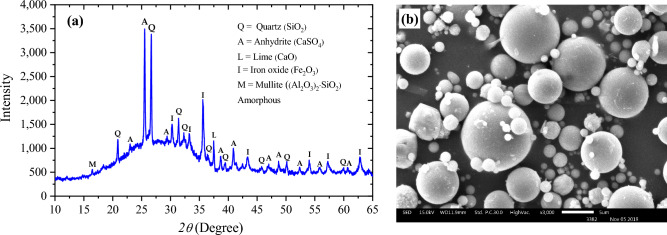
(**a**) XRD patterns and (**b**) SEM image of FA.

**Figure 3:**
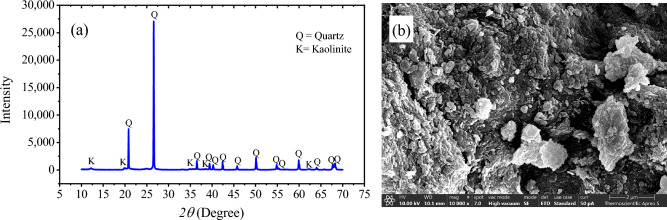
(**a**) XRD patterns and (**b**) SEM image of loess.

Based on the XRD patterns shown in Fig. 2, the primary crystalline minerals identified in FA are quartz, anhydrite, ferric oxide, and lime. Additionally, amorphous phases were observed, indicated by the dispersion peaks between 15° and 30°. The Rietveld refinement results investigated that FA contains 57.16 wt% amorphous phases, which might indicate high pozzolanic activity^30^. The morphology of the FA particles was determined with a scanning electron microscope (SEM), and the results are presented in Fig. 2b. The SEM micrograph revealed that all the FA particles had a spherical shape, with a broad range of particle sizes ranging from the nanoscale to larger than 10 microns. The present study utilized natural loess soil obtained from Khon Kaen University, Thailand. The grain size distribution and consistency test results revealed that this soil is classified as silty sand corresponding to the USCS classification system. The soil was sieved through a 2 mm sieve before being used. The XRD patterns of loess soil are shown in Fig. 3a. The predominant component of the loess soil is quartz. This finding is consistent with the results of the chemical composition analysis. Figure 3b displays an SEM photo of the original loess, which clearly illustrates the shape of the soil particles. The image indicates that the loess soil is composed of several small particles with a spherical shape that join together with weak agglomeration to form larger particles.

### Mixture design and sample preparation for paste and mortar

To determine the optimal mixing ratio of CCR to FA, the chemical composition of the raw materials was considered, which is highly influential on the rate of pozzolanic reaction in the mixture. The optimal molar ratio of CaO/(SiO_2_ + Al_2_O_3_) for the pozzolanic reaction between amorphous siliceous or aluminous materials and calcium hydroxide in the presence of water were also study referred to Eqs. (1) and (2) ^31,32^. These equations suggest that the theoretical ratio of CaO/(SiO_2_ + Al_2_O_3_) required to complete the pozzolanic reaction is 1.5. Subsequently, we utilized the content of CaO, SiO_2_, and Al_2_O_3_ in the raw materials to derive an empirical formula for the optimized CCR:FA ratio, which we computed to be 0.81. However, several investigations have suggested that the pozzolanic reactions between Ca(OH)_2_ with SiO_2_ and Al_2_O_3_ are very complicated^33–38^. Therefore, this investigation designed the raw material ingredient with a CaO/(SiO_2_ + Al_2_O_3_) ratio in the range of 1.7 to 0.9. The specific mixture proportions for the pastes can be found in Table 3.

**Table 3:** Mixture design for pastes with different CCR/FA ratios.

Mixture ID	CaO/(SiO_2_ + Al_2_O_3_)	CCR (wt%)	FA (wt%)	CCR/FA
C-1.7	1.7	45	55	0.81
C-1.5	1.5	41	59	0.69
C-1.3	1.3	36	64	0.57
C-1.1	1.1	31	69	0.46
C-0.9	0.9	26	74	0.34

One aspect of significance in this study is the estimation of the optimal CCR to FA mixing ratio, which was predicated on the chemical composition of the materials together with the characteristics of pozzolanic reactions. However, it is pertinent to acknowledge that the pozzolanic reactions, as shown in Eqs. (1) and (2), depend on the amorphous SiO_2_ and Al_2_O_3_ from FA, amounting to merely 57.16 wt%. This indicates that not all SiO_2_ or Al_2_O_3_ are involved in the reactions. In a similar vein, only a fraction of CaO from CCR, specifically the portlandite phase constituting 32.02 wt%, undergoes reaction. Therefore, an approach that determines the optimal mixing ratio from the amorphous phase content in FA and the portlandite phase content in CCR may offer a more reasoned basis and should be contemplated for estimating the optimal mix. Nonetheless, the analysis of the amorphous phase content in raw materials through the Rietveld refinement technique, derived from X-ray diffraction patterns, presents a considerably complex process. This method necessitates specialized expertise, sophisticated tools, and advanced techniques. Considering these considerations, the present study elected to establish the optimal ratio of raw materials based on their chemical composition to facilitate user convenience. The optimal CCR to FA mixing ratio, calculated with a focus on the reactive phases, the amorphous phase in FA and portlandite in CCR, was ascertained based on the siliceous pozzolanic reaction (Eq. (1)). The calculation result revealed that the optimal CCR/FA ratio is 3.30. This ratio stands in contrast to the experimental design implemented in this research, as detailed in Table 3. This divergence is a point of considerable interest and necessitates extensive investigation into future work.

The CCR and FA dry ingredients were mixed using the ball-milling method at low speed for 5 min until a well-mixed powder was obtained. The raw materials were then transferred to a mixer, and the paste was mixed following the ASTM C 305-20 standard^39^. The water-to-binder ratio was fixed at 0.65 for all compositions. The fresh pastes were cast into 25-mm cube molds and kept at room temperature of 25 ± 1 °C with relative humidity > 95%) for 24 h before being removed from the molds. The demolded samples were then placed in a curing room with a relative humidity exceeding 60% until achievement of designated test age.

1 $$3{\text{Ca}}({\text{OH}})_{2} + 2{\text{SiO}}_{2} \to 3{\text{CaO}} \cdot 2{\text{SiO}}_{2} \cdot 3{\text{H}}_{2} {\text{O}}$$

2 $$3{\text{Ca}}({\text{OH}})_{2} + {\text{Al}}_{2} {\text{O}}_{3} { } + { }3{\text{H}}_{2} {\text{O}} \to 3{\text{CaO}} \cdot {\text{Al}}_{2} {\text{O}}_{3} \cdot 6{\text{H}}_{2} {\text{O}}$$

The specific mixture proportions for the mortar samples can be found in Table 4. The ratio of CCR/FA is fixed in a manner similar to the paste mixture design. Each specimen was prepared with a constant water-to-binder ratio (*w/b*) of 0.58 and a sand-to-binder ratio of 2.75.

**Table 4:** Details of mortar mix compositions*.*

Mixture ID	OPC (kg/m^3^)	CCR (kg/m^3^)	FA (kg/m^3^)	Sand (kg/m^3^)	Water (kg/m^3^)	SP (kg/m^3^)	Flow (%)
Control	500			1375	290	2.9	112
C-1.7		223	277	1375	290	5.6	110
C-1.5		204	296	1375	290	4.8	107
C-1.3		182	318	1375	290	4.6	110
C-1.1		157	343	1375	290	3.9	109
C-0.9		128	372	1375	290	2.1	111

It should be noted that the *w/b* used in this study is slightly higher than the recommended ratio of 0.485, as per ASTM C109 standard^40^. This is due to the fact that, although the river sand used in this work is natural silica sand conforming to the requirements of the graded standard sand in ASTM C778 standard^41^, it exhibits higher water absorption and a more angular shape compared to Ottawa sand, resulting in a higher water demand. However, it should be acknowledged that an excess of water added at any time will result in free water being trapped in the concrete or rising to the top, causing bleeding. To achieve the desired workability, a type F superplasticizer was introduced to ensure a flowable consistency within the range of 110 ± 5%. Mortar mixtures were produced as 50 mm cubes in accordance with the specifications of the ASTM C305-20 standard. The OPC mortar was created and designated as the control mixture to evaluate the mechanical properties of CCR-FA binders.

### Evaluation of paste and mortar samples

#### Mechanical properties

The compressive strength of the CCR-FA binders was measured at curing ages of 7, 14, 28, 56, and 91 days. For each age, three 25 mm cubes of paste and three 500 mm cubes of mortar were selected and tested using a compressive testing machine. The testing procedure was carried out according to the ASTM C109 standards^40^. The applied load speed was controlled at 50 N/s and 900 N/s for paste and mortar, respectively.

#### Phase formation and microstructure characterization

In support of the mechanical property evaluation, phase formation and microstructural investigations were conducted via the X-ray diffraction (XRD) method and scanning electron microscopy (SEM), respectively. X-ray powder diffraction patterns were obtained using Cu-Kα radiation (λ = 1.5406 Å) radiation on a D Bruker, D2 PHASER. The diffraction pattern (2*θ*) was scanned over 5°–70°, with a step size of 0.02 and a time per step of 0.5 s. For SEM analysis, a field emission scanning electron microscope (Thermofisher, Aspero S) equipped with energy-dispersive X-ray spectroscopy (EDS) is operated at a working distance of approximately 10 mm and an accelerating voltage of 5.0–10 kV. For the SEM-based microstructural analysis, the hardened pastes were initially reduced to particles within a size range of 2–5 mm. Subsequently, immersion in acetone for a duration of 24 h was employed to ensure the removal of any residual free water, following the methodology outlined by Uzal et al.^42^. Thereafter, the samples underwent a meticulous vacuum drying process at 60 °C for 24 h, to prepare them adequately for subsequent testing procedures.

### Sample preparation and testing program for soil stabilization

**Figure 4:**
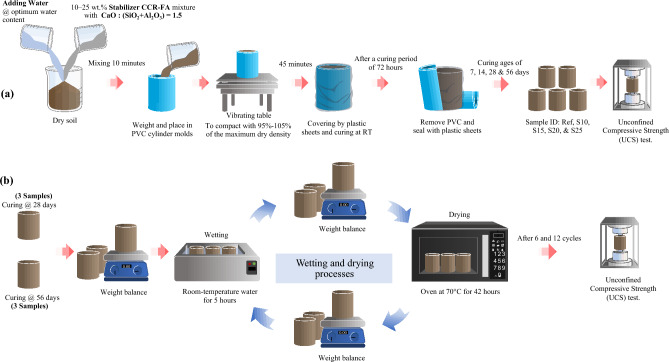
(**a**) Preparation processes for stabilized soils and the testing for UCS and (**b**) Testing program for mass loss and strength loss of stabilized soils under wetting and drying processes.

The CCR-FA mixture with the optimal CaO/(SiO_2_ + Al_2_O_3_) composition, which exhibited the highest mechanical performance for both paste and mortar, was chosen as the binder to improve the engineering properties of the loess soil. The mixture of CCR-SF, which provided the highest binding performance from paste and mortar test results, was employed as a stabilizer for 10–25 wt% of the dry soil. The stabilizer, dry soil, and water at optimum water content were well mixed for 10 min by the milling machine (Table 5). After mixing, the fresh mixture was weighed and placed in PVC cylinder molds with a diameter of 50 mm and a height of 100 mm and compacted on a vibrating table for 45 min with a density in the range of 95% to 105% of the maximum dry density. The molded samples were covered with plastic sheets to retain moisture and left to cure at room temperature. After a curing period of 72 h, the samples were taken out of the molds and then sealed with plastic sheets until the required curing times for testing. The unconfined compressive strength (UCS) test was carried out using a compression machine with a vertical deformation rate of 1.0 mm per minute. To ensure the reliability of the results, three samples of each mixture were prepared and tested under identical conditions at curing ages of 7, 14, 28, and 56 days. The preparation processes of the samples and the testing for UCS are illustrated in Fig. 4a.

**Table 5:** Mixture proportions for soil stabilization.

Mixture ID	Loess (kg/m^3^)	CCR (kg/m^3^)	FA (kg/m^3^)	Water (kg/m^3^)	Maximum dry density (kg/m^3^)
Ref	2160	–	–	203	1980
S10	1944	89	127	203	1987
S15	1836	133	191	203	1989
S20	1728	177	255	203	1981
S25	1620	221	319	203	1987

Loess soil is highly vulnerable to changes in moisture content, which can significantly impact its strength and deformation behavior. As such, this study aimed to investigate the mass and strength loss resulting from wetting and drying cycles. The method for determining mass loss due to wetting and drying conditions, along with strength loss, was adopted from the ASTM D559 standard^43^. Three samples were cured for a specified curing period, and their original masses were recorded. The samples were then immersed in room-temperature water for 5 h. Following this soaking period, the samples were removed, and their masses were recorded. The samples were then moved to an oven and heated at 70 °C for 42 h before being weighed again. This entire process constituted one cycle of the wetting and drying test, and the testing continued until the completion of the twelfth cycle. To accurately assess the performance of stabilizers in conditions specific to Thailand, a tropical country with frequent rainfall throughout most of the year, a carefully considered curing period is essential. The curing period before exposure to a wet-dry cycle environment should extend beyond 28 days. This duration is necessary to allow adequate reaction time for the stabilizer to achieve its intended effect. However, this period should not exceed 60 days, aligning with the typical dry spell duration in Thailand. This recommendation is in accordance with the comments provided by the Department of Highways (DOH), Thailand. Hence, the testing was conducted on samples that had been cured for 28 days and 56 days. The UCS test on the samples subjected to wetting and drying processes was performed at the end of the first, sixth, and twelfth cycles. The testing program for mass loss and strength loss of stabilized soils under wetting and drying processes is detailed in Fig. 4b.

## Results and discussion

### Compressive strength of past and mortar

**Figure 5:**
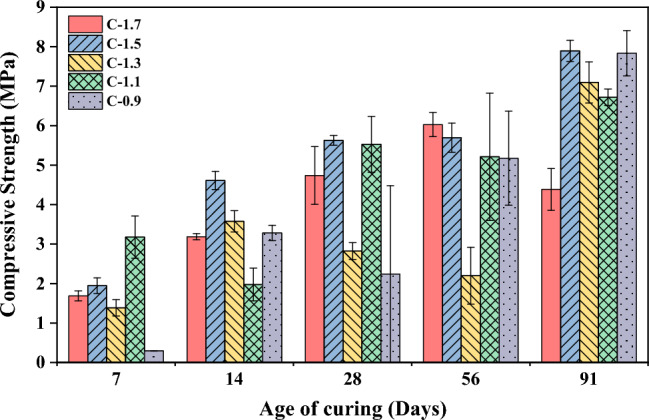
Age-dependent compressive strength development of paste.

**Figure 6:**
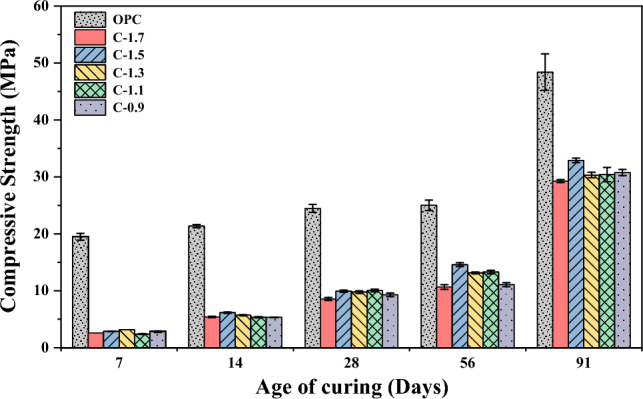
Age-dependent compressive strength development of mortar.

**Figure 7:**
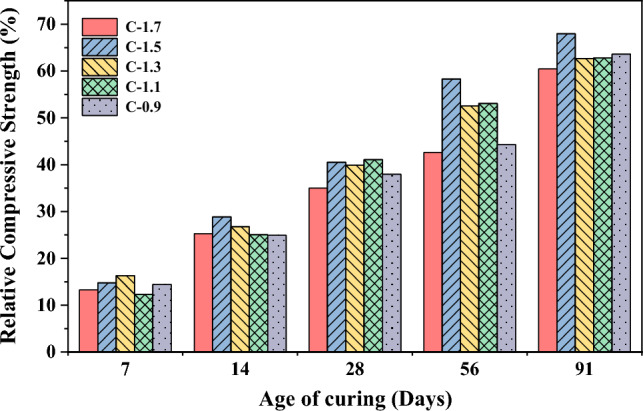
Relative Compressive strength of mortar.

In Fig. 5, the development of compressive strength in CCR-FA pastes is presented for several curing ages. At 7 days of curing, the compressive strength of pastes ranged from 1–2 MPa, except for the C-0.9 mixture, which showed a compressive strength lower than 0.5 MPa. The compressive strength of all mixture compositions tended to increase continuously with an increase in curing age. The C-1.5 mixture displayed the highest compressive strength at a long curing period, with a strength value of 7.89 MPa at 91 days of curing. However, the strength development of CCR-FA binders over time showed an abnormal trend for some mixture compositions, as the compressive strength may either increase or decrease with an increase in curing period. The compressive strength characteristics of the CCR-FA binder were investigated for mortar samples using OPC as the reference binder, as illustrated in Fig. 6. The results indicate that the compressive strength increases with an increase in curing age for all mixtures. To compare the compressive strength of CCR-FA mortar with that of OPC, the relative compressive strength was calculated and plotted in Fig. 7. At the 7-day curing age, the compressive strength of CCR-FA mortar exhibited a notable reduction compared to that of OPC mortar, with a relative compressive strength of less than 20% for all mixtures. This indicates that the pozzolanic reactions occurring in the CCR-FA binder generate reaction products slowly, which gradually increase over time. After 91 days, the relative compressive strength of CCR-FA binders improved to higher than 60% for all mixtures. The highest relative compressive strength of 68% compared to OPC mortar was observed in the C-1.5 mixture, which corresponds with the compressive strength test results for paste. These findings suggest that CCR-FA binder can be a viable alternative to OPC binder in mortar, particularly for long-term applications where strength development over time is crucial.

### Microstructural analysis

**Figure 8:**
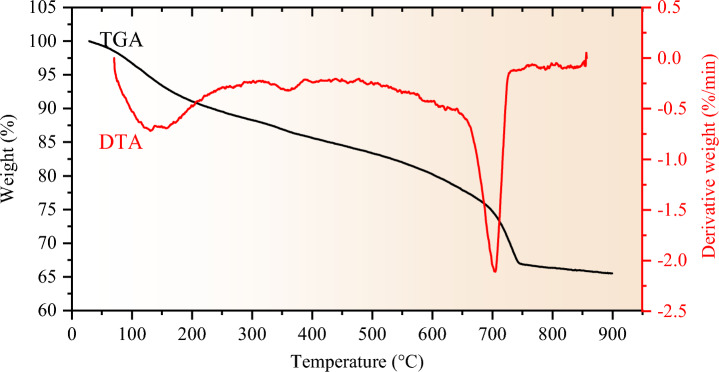
TGA-DTA curves of C-1.5 paste after 56 days of curing.

The TGA-DTA facilitates a preliminary identification of the reaction mechanisms in the CCR-FA paste. Figure 8 presents the TGA-DTG curves of the CCR-FA paste after a curing period of 56 days. At temperatures below 120 °C, the weight loss observed is primarily due to the evaporation of free water from the sample. Beyond this temperature threshold, the results reveal three major stages of weight loss. The first stage involves the dihydroxylation of hydrated components in CHS gels, ettringite, and AFm phases, which occurs between 150 °C and 400 °C. The second stage, evident between 420 °C and 500 °C, corresponds to the dehydration of Ca(OH)_2_. The final stage, occurring between 550 °C and 800 °C, is marked by the decomposition of calcite (CaCO_3_) in the raw materials, resulting in the formation of CaO and CO_2_. These test results provide insights into the potential phases identifiable in the hydrated pastes.

**Figure 9:**
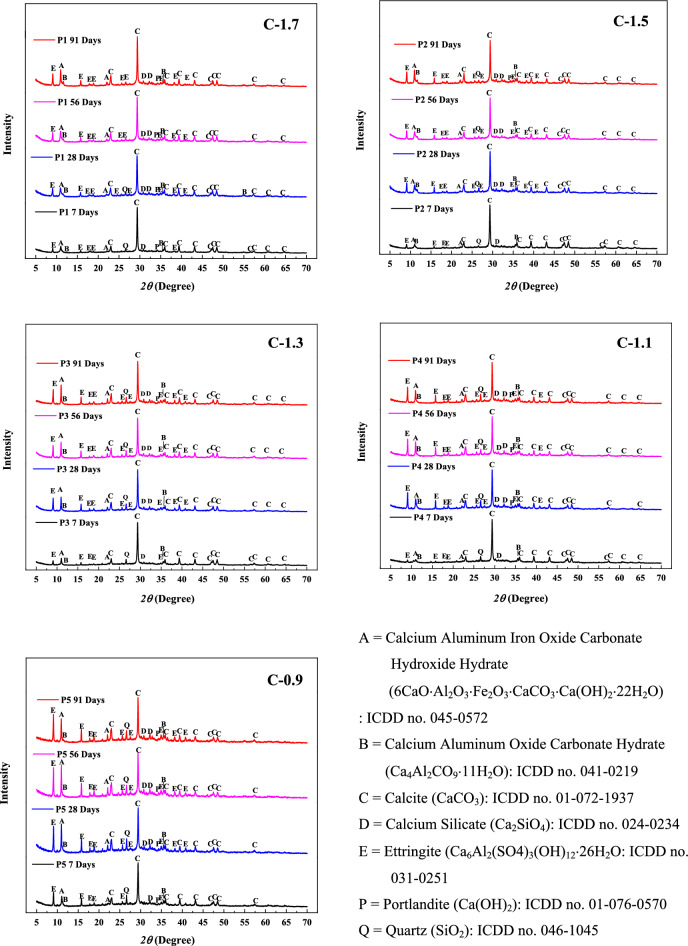
XRD Patterns of CCR-FA pastes with different compositions.

The mineralogical analysis of hydrated pastes, formulated from CCR and FA mixtures with varying CaO/(SiO_2_ + Al_2_O_3_) ratios, was conducted at different curing ages using the X-ray diffraction (XRD) method (Fig. 9). The results revealed that the diffraction peak characteristics exhibited slight variations due to differences in the mixture proportions. One notable observation from the XRD patterns was the presence of calcite, which is a residue phase originating from the CCR raw material. In addition to calcite, several hydrated phases were identified as products of the reactions between the raw materials. These phases included calcium aluminum iron oxide carbonate hydroxide hydrate (6CaO·Al_2_O_3_·Fe_2_O_3_·CaCO_3_·Ca(OH)_2_·22H_2_O, ICDD no. 045-0572), calcium aluminum oxide carbonate hydrate (Ca_4_Al_2_CO_9_·11H_2_O, ICDD no. 041-0219) and ettringite (ICDD no. 041-0219). As the curing age increased, a significant enhancement in the peak intensity of these hydration products was observed. This effect was particularly evident in samples with lower CaO/(SiO_2_ + Al_2_O_3_) ratios. The intensity peaks of ettringite, relative to calcite, were higher in samples with low CaO/(SiO_2_ + Al_2_O_3_) mixtures compared to those with high CaO/(SiO_2_ + Al_2_O_3_) ratios. This outcome can be attributed to the higher content of fly ash in the low CaO/(SiO_2_ + Al_2_O_3_) samples, which serves as the raw material responsible for generating the ettringite phase.

**Figure 10:**
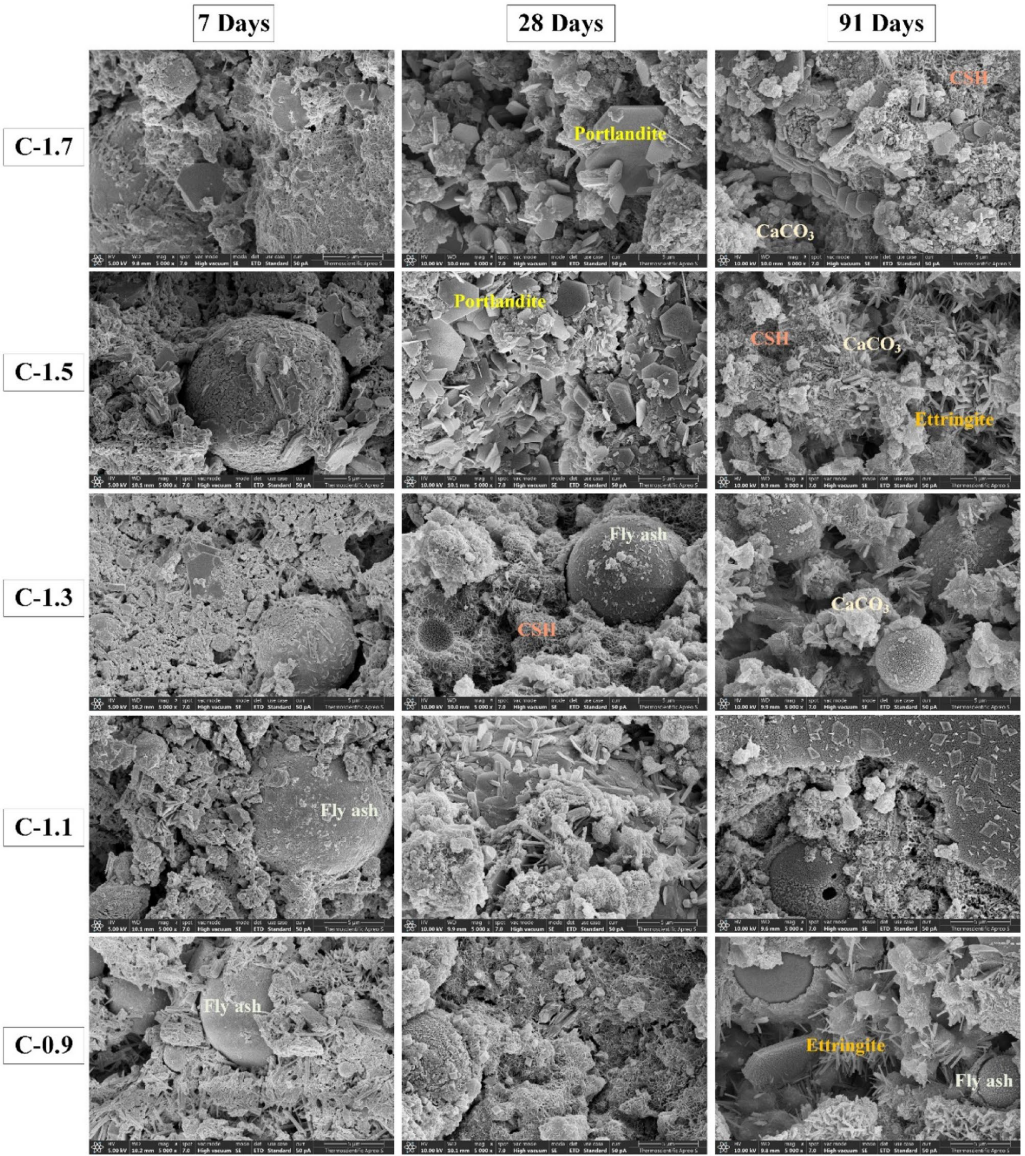
SEM morphology of CCR–FA pastes with different CaO(SiO_2_ + Al_2_O_3_) ratios.

**Figure 11:**
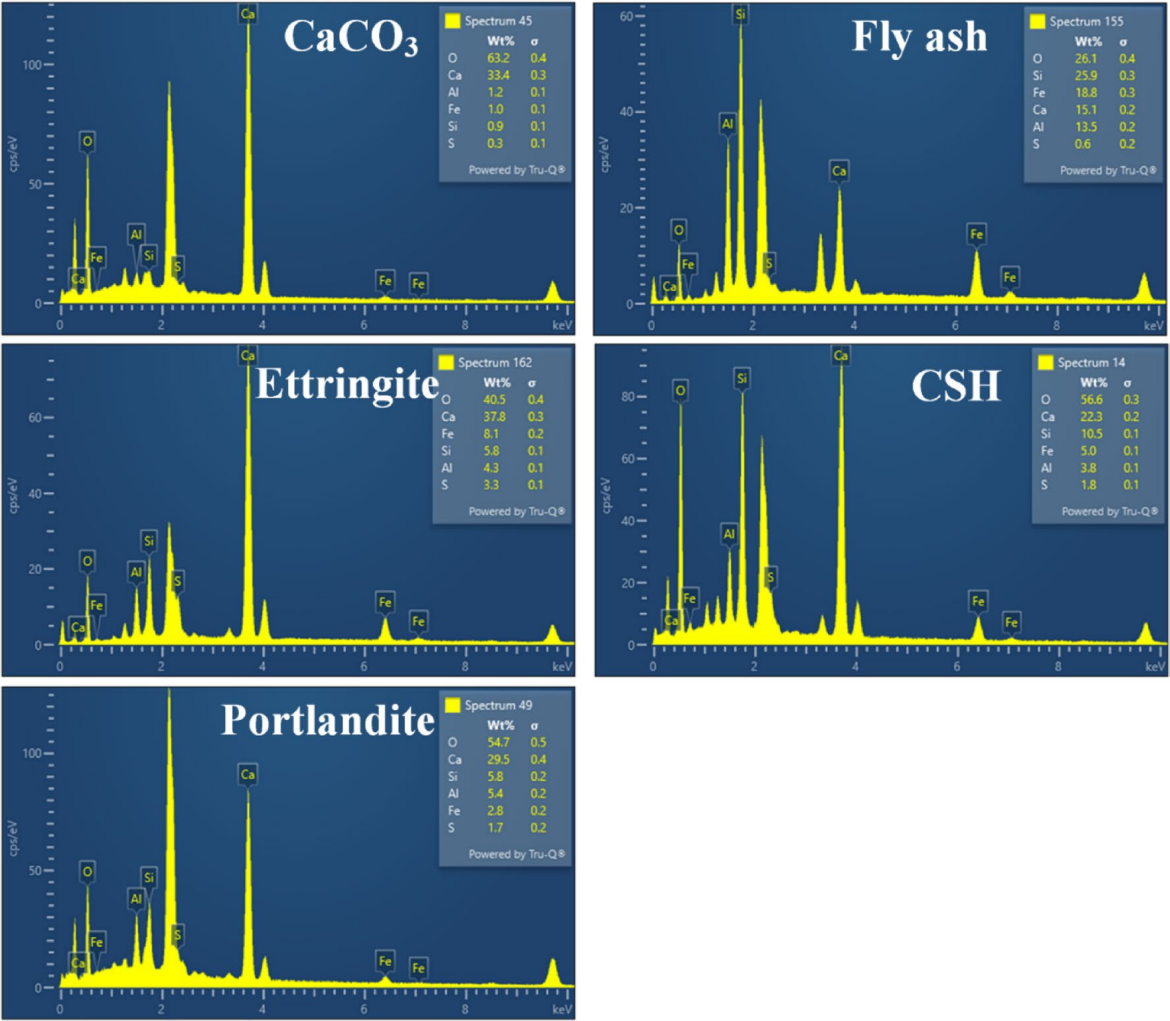
Energy dispersive spectroscopy analysis on CCR-FA pastes.

The results of SEM–EDS analysis on CCR-FA pastes with different CaO(SiO_2_ + Al_2_O_3_) ratios and curing periods are presented in Figs. 10 and 11, respectively. The primary cementing agent in the hydration products, Calcium Silicate Hydrate (CSH), was identified by its Ca/Si atomic ratio obtained from EDS analysis. However, several previous studies examining the nanoscale structure of C–S–H suggest that there are various CSH structures, with defective SiO_4_ tetrahedra chains resulting in a broad Ca/Si ratio ranging from 0.83 to 2.25^44^. Therefore, the identification of CSH using the SEM–EDS technique is only a rough estimation due to the highly complex and potentially defective structure of CSH. The hydration products of C-1.7 and C-1.5 pastes, which had a high content of CCR powder, consisted mainly of plate-like portlandite after 28 days of curing. Nonetheless, extending the curing duration to 91 days led to a reduction in portlandite crystals, which were replaced by CSH and CaCO_3_ due to the pozzolanic and carbonization reactions. For the C-1.3, C-1.1, and C-0.9 pastes, which had a rich content of fly ash in their raw materials, unreacted fly ash was clearly observed. Several types of hydration products were observed, with CSH being the primary cementing agent formed from the hydration reactions of the raw materials, alongside ettringite, which is present but does not contribute to cementing ability. These results were consistent with the XRD investigation results. The microstructural investigation results suggest that the use of CCR and fly ash as raw materials can lead to the formation of various types of hydration products, which can enhance the strength and durability of the resulting reaction products.

### Soil stabilization

**Figure 12:**
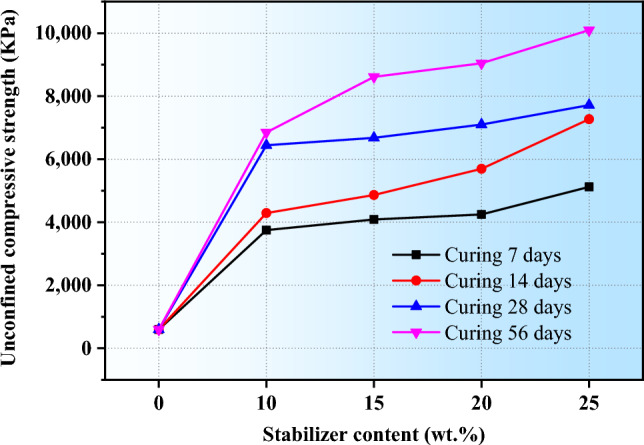
The correlation between the UCS and the fraction of stabilizer under different curing ages.

Figure 12 illustrates the variation in the UCS of loess soil stabilized with a CCR-FA, at a CCR -FA ratio of 0.69, across various curing periods and stabilizer contents. The UCS of the stabilized soils increased in a nonlinear manner with the duration of the curing periods. In the absence of the stabilizer, the UCS of the loess remained at 595 kPa. However, with the addition of a 10 wt% CCR-FA stabilizer, the UCS reached 3751, 4289, 6441, and 6848 kPa after curing periods of 7, 14, 28, and 56 days, respectively. These values were approximately 6, 7, 11, and 12 times higher than those of the unstabilized soil, demonstrating a remarkable improvement. The influence of the curing duration on the strength of the stabilized loess soils was found to be dependent on the quantity of stabilizer added. A slight improvement in strength was observed during the 7-days to 14-days curing period when a low stabilizer content was used, while a significant increase was observed between the 14-days to 28-days curing period. Notably, when the stabilizer content was increased to 25 wt%, a substantial enhancement in strength was observed between the 14-days and 28-days curing periods. Furthermore, a significant improvement in the UCS of the stabilized loess was observed for longer curing periods, specifically from 28 to 56 days, with an increase in the stabilizer content. These results suggest that a higher content of CCR-FA stabilizer is more effective for longer curing periods due to the prolonged pozzolanic reaction required for the generation of the cementitious binder.

**Figure 13:**
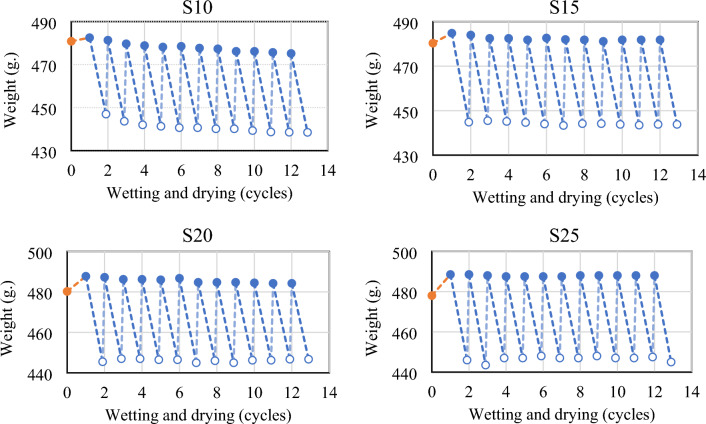
Variation in mass resulting from wet-dry cycles of stabilized soil with cured at 28 days before testing.

**Figure 14:**
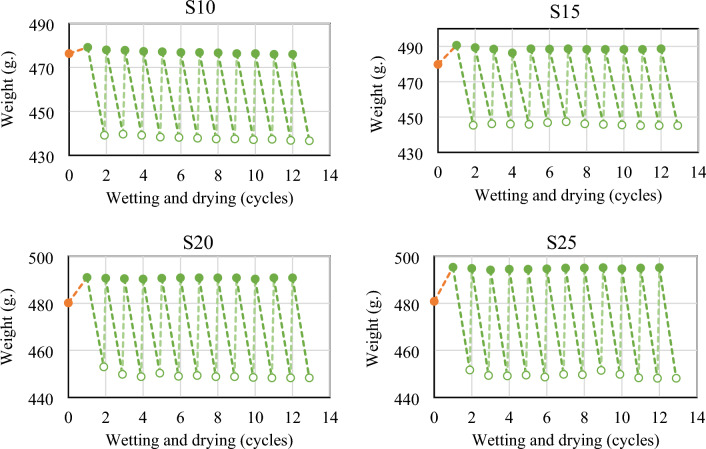
Variation in mass resulting from wet-dry cycles of stabilized soil with cured at 56 days before testing.

The results of the wet-dry durability tests, represented by mass losses after each cycle, were graphed against the curing durations for all stabilized soil combinations. These results are displayed in Figs. 13 and 14. It was found that samples stabilized with a CCR-FA mixture stabilizer significantly maintained their original weight and survived a full 12 cycles of wet–dry testing. In the case of a 10 wt% stabilizer addition, the mass of samples gradually decreased with each wetting and drying cycle, but the samples still retained more than 90 wt% of their original mass after 12 cycles (refer to Table 6). The study found that increasing the content of CCR-FA stabilizer by 15–25 wt% led to an improvement in the rate of leaching from wetting and drying cycles. This improvement can be attributed to the larger content of cementitious agents resulting from the pozzolanic reaction of the stabilizer. The results, as shown in Fig. 13, indicate that samples subjected to a longer curing period of 56 days demonstrated superior performance and survived a full 12 cycles of wet-dry testing.

**Table 6:** Residue mass percentage of stabilized soil after wetting and drying cycle.

	S10		S15		S20		S25	
	28 days	56 days	28 days	56 days	28 days	56 days	28 days	56 days
Original mass (wt%)	100.00	100.00	100.00	100.00	100.00	100.00	100.00	100.00
Residue mass after 6 wetting–drying cycles (wt%)	91.65	91.92	92.30	93.23	92.66	93.58	93.52	93.57
Residue mass after 12 wetting–drying cycles (wt%)	91.20	91.67	92.40	92.78	93.02	93.37	93.10	93.47

**Figure 15:**
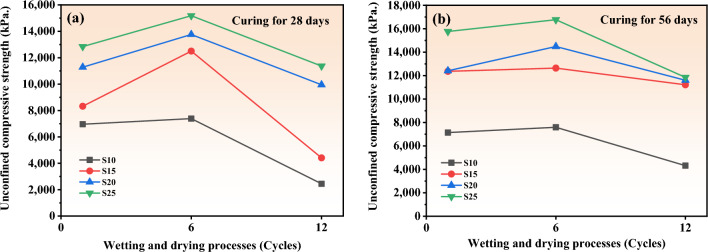
The UCS stabilized soil after wetting and drying processes.

**Figure 16:**
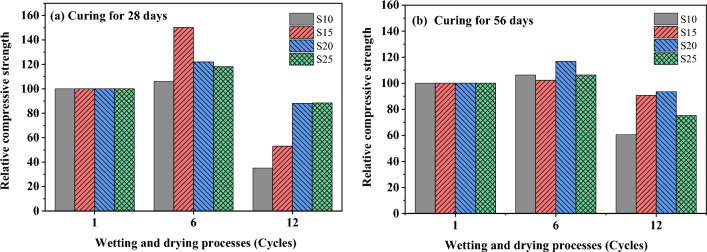
Relative compressive strength of stabilized soil after wetting and drying processes.

Figure 15 illustrates the evolution of UCS with the number of wet-dry cycles for samples tested in this study. The UCS initially increased with the number of wet-dry cycles up to 6, compared to their residual strength. However, the UCS begins to decrease when the number of wetting and drying cycles reaches 12. The impact of wet-dry cycles on UCS development in stabilized soils was observed to be similar in several previous studies, where UCS increased initially with the number of cycles and then gradually decreased as the cycles continued^45–47^. These previous studies suggested increasing the curing temperature during the drying phase of stabilized materials can accelerated the reaction rate of stabilizers and enhance the strength of the mixtures. The relative UCS demonstrates a significantly higher increase in compressive strength for soils stabilized and cured for 28 days, compared to those cured for 56 days, before the wet-dry cycles test (Fig. 16). This can be attributed to the fact that the original UCS of the soils stabilized and cured for 56 days is significantly higher than that of the samples cured for 28 days, due to the longer curing period. The decrease in UCS after longer wet-dry cycles is attributed to the repeated drying-wetting process, which induces microcracks and compromises the integrity of the specimen^48^. This allows water to infiltrate along the cracks, leading to a reduction in UCS. Moreover, samples cured for 56 days exhibit a smaller decrease in strength due to prolonged wet-dry cycles compared to samples cured for 28 days (Fig. 16). This suggests that a longer curing period, which generates more cementing materials, helps to improve the durability of stabilized soils under a wet-dry cycle environment. From a mechanical perspective, the shear strength of soils is composed of two primary components: cohesion and the internal friction angle. Previous studied have demonstrated that during dry–wet cycles, water predominantly disrupts the bonds between soil particles, leading to a reduction in cohesion. However, the process of dry–wet cycling has minimal impact on the roughness of soil particles, and as a result, the internal friction angle remains relatively unchanged^47,49^.

However, the significantly reduction in UCS of stabilized soil with high stabilizer content of 25 wt% (S25) cured for 56 days after 12 cycles of wet–dry testing is doubtful. This may be high cementing agent generated from stabilizer loss their bonding ability after longer cycle of wet-dry environment and resulting in significantly reduction in cohesion. However, deep investigation on this point need to more clarify. The notable decrease in the UCS of the stabilized soil sample with a high stabilizer content of 25 wt% (S25), cured for 56 days following 12 cycles of wet-dry testing, raises questions. This reduction may be attributed to the high concentration of cementing agents from the stabilizer losing their bonding effectiveness after prolonged exposure to wet-dry cycles, leading to a significant decrease in cohesion. However, this hypothesis needs further detailed investigation to elucidate the underlying mechanisms and confirm the observed trend.

## Conclusion

This study explores the development of calcium carbide residue (CCR) and fly ash (FA) as alternative binders, proposing them as sustainable options alongside ordinary Portland cement for loess soil stabilization. X-ray diffraction analysis indicates that raw FA contains 57 wt% of amorphous phase content, while CCR consists of 66 wt% calcite and 32 wt% portlandite. The CCR-FA mixture with a Ca/(SiO_2_ + Al_2_O_3_) ratio of 1.5 yielded the highest compressive strength over extended curing periods. However, further investigation is needed to optimize the mixture proportions and understand their long-term behavior under various environmental conditions. The application of the CCR-FA mixture in soil stabilization, particularly with a Ca/(SiO_2_ + Al_2_O_3_) ratio of 1.5, demonstrated significant improvements in unconfined compressive strength. The soils stabilized with the CCR-FA binder exhibited an initial enhancement in unconfined compressive strength, observable through up to six wet-dry cycles. This increase is likely attributable to the synergistic physical and chemical reactions within the soil. However, extending the wet-dry cycles to twelve led to a reduction in strength, presumably due to microcrack formation induced by these repeated cycles.

## Data Availability

The research data used to support the finding of this study are described and included in the article. Furthermore, some of the data used in this study are also supported by providing references as described in the article.
